# Levofloxacin-Induced Generalized Fixed Drug Eruption

**DOI:** 10.7759/cureus.86372

**Published:** 2025-06-19

**Authors:** Hiebda Sofía Martínez Jiménez

**Affiliations:** 1 Internal Medicine, Centro Médico Nacional Siglo XXI, Mexico City, MEX

**Keywords:** adverse drug reactions, dermatology, drug eruptions, drug hypersensitivity, levofloxacin, quinolones, skin disease

## Abstract

Fixed drug eruption (FDE) is a type of toxicodermatitis induced by a delayed hypersensitivity reaction (type IV) characterized by the reappearance of erythematous-violaceous plaques at the same site upon reexposure to the triggering agent. We present the case of a 72-year-old female who developed a generalized FDE following the administration of levofloxacin. Diagnosis was established clinically and supported by the Naranjo algorithm. The condition improved upon drug discontinuation. This case highlights the importance of recognizing less common presentations, such as generalized FDE, and the need for continued awareness among healthcare providers, especially given the frequent use of antibiotics like levofloxacin.

## Introduction

Adverse drug reactions are typically classified into two types: dose-dependent reactions, which are predictable and based on the drug’s pharmacological properties, and dose-independent reactions, which are unpredictable and influenced by both individual patient factors and the drug itself. Drug eruptions generally fall into the latter category and account for approximately 25-30% of all adverse drug reactions [1]. Fixed drug eruptions (FDE) account for 14-22% of all cutaneous drug reactions and occur after morbilliform and urticarial drug reactions. Nonsteroidal anti-inflammatory drugs (NSAIDs) and antibiotics are most commonly responsible for causing FDE [2,3].

## Case presentation

A 72-year-old female with a history of chronic heart failure and chronic coronary syndrome (CCS II, clinical stage IV), both of which had remained untreated by the patient's choice, presented to the emergency department of our unit. The medical history included an ileostomy following a complicated appendectomy, which was managed with right hemicolectomy and ileostomy. The patient had no known allergies to medications.

The patient had a clinical presentation of moderate dehydration, accompanied by asthenia, adynamia, fatigue, and a cough that produced white sputum. During the first 24 hours of hospitalization, the patient developed hypotension and fever. Chest radiography revealed a consolidation in the left lower lobe. Given the clinical scenario suggestive of pulmonary infection, the medical team initiated empirical antibiotic therapy with a respiratory quinolone (levofloxacin).

Within eight hours of drug initiation, the patient developed disseminated dermatitis involving the chin and both upper and lower extremities. The lesions were characterized by multiple oval erythematous-violaceous plaques with well-defined borders and laminated surface scaling. The lesion sizes varied, with the largest measuring 10 × 5 cm² on the right thigh. Approximately twelve lesions were identified. No orogenital lesions were observed.

**Figure 1:**
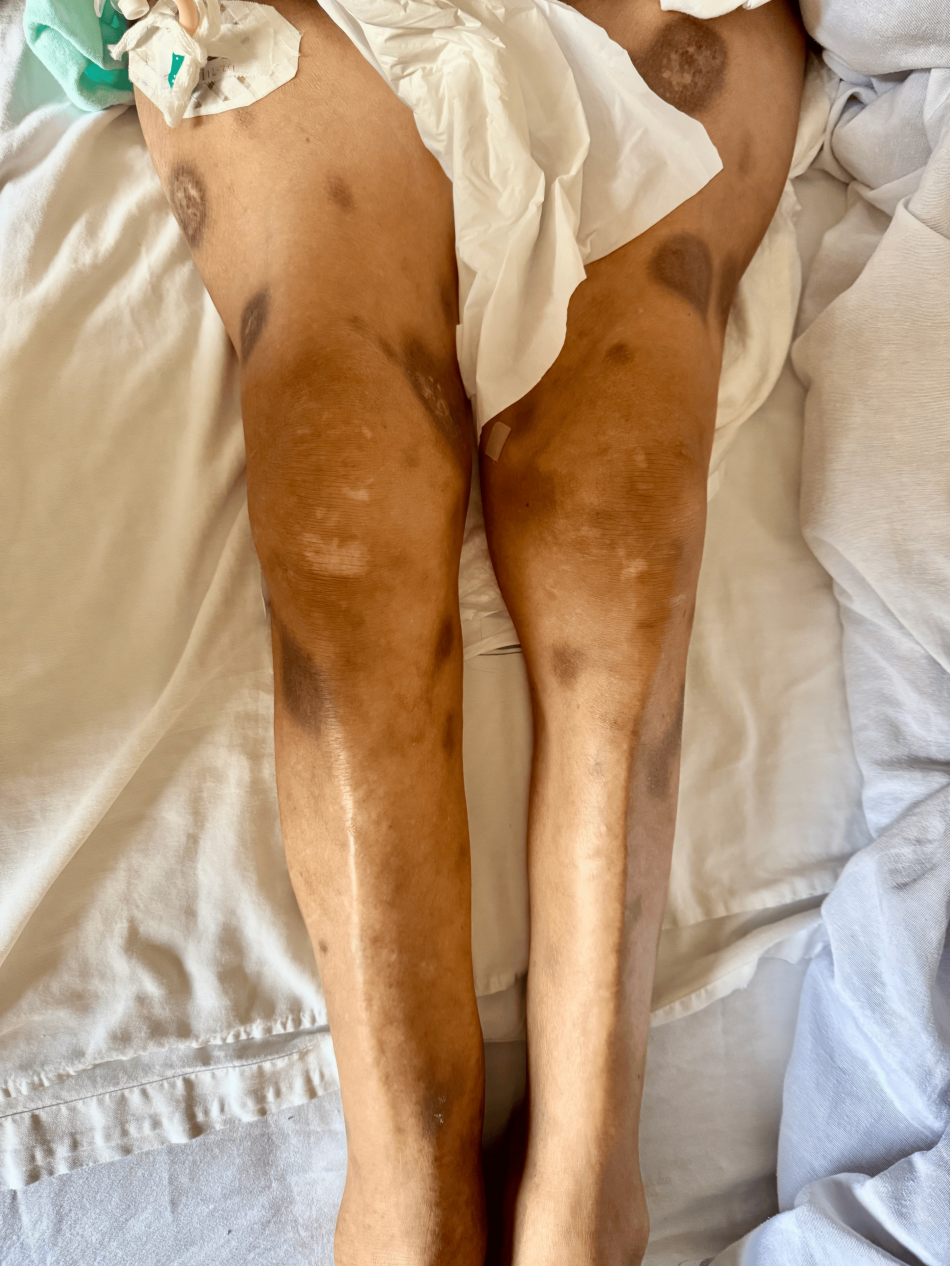
Post-inflammatory hyperpigmentation involving the lower extremities

**Figure 2:**
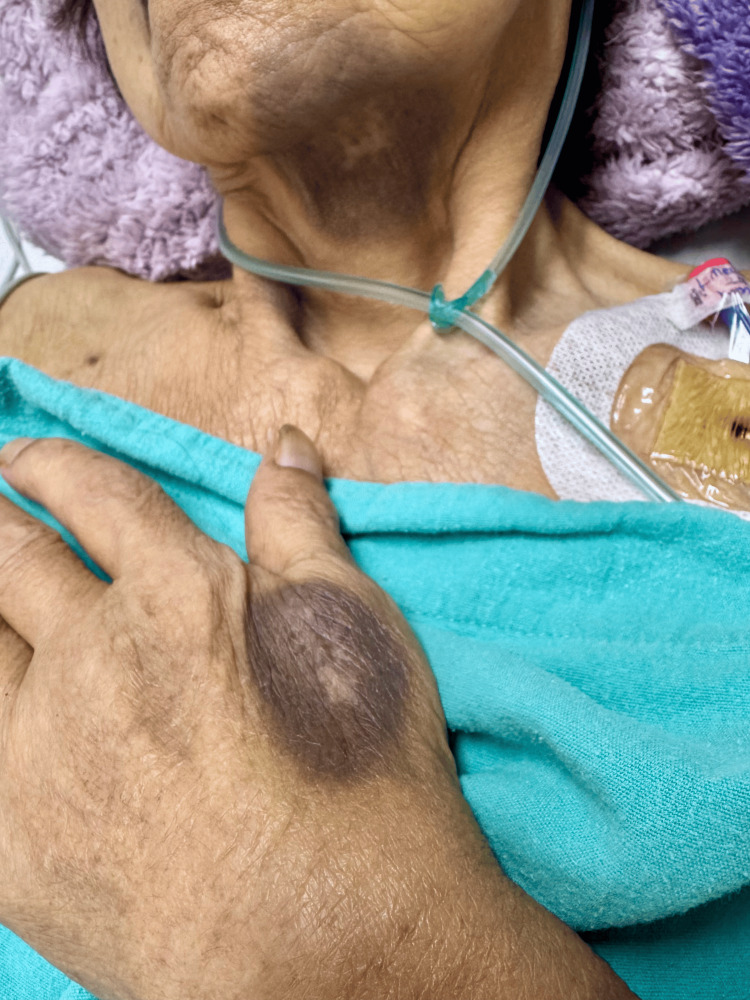
Post-inflammatory hyperpigmentation involving the chin and hand

The patient denied recent exposure to other medications. Based on the clinical findings, a diagnosis of generalized FDE secondary to levofloxacin was established. Therefore, levofloxacin was immediately discontinued. After suspension, the skin lesions began to fade, resulting in residual post-inflammatory hyperpigmentation (Figures 1-2).

The patient declined to undergo confirmatory testing. Therefore, we used the Naranjo algorithm [4] to determine the causal relationship between the drug exposure and adverse reaction and obtained a score of 6. The adverse reaction was considered "probable" based on the following criteria: "The adverse event appeared after the suspected drug was administered," "The adverse reaction improved when the drug was discontinued," and "There were no alternative causes (other than the drug) that could cause the reaction" (Table 1) [4]. Topical treatment with conventional emollients was initiated, resulting in significant clinical improvement.

**Table 1:** Adverse drug reaction probability scale applied to the present case Based on the patient’s responses, a total score of 6 was obtained. According to the original classification system by Naranjo et al. (1981), this corresponds to a “probable” adverse drug reaction (definite ≥9, probable 5–8, possible 1–4, doubtful ≤0) [4].

	Yes	No	Score
Are there previous conclusive reports on this reaction?	+1	0	+1
Did the adverse event appear after the suspected drug was administered?	+2	-1	+2
Did the adverse reaction improve when the drug was discontinued or a specific antagonist was administered?	+1	0	+1
Did the adverse reaction reappear when the drug was readministered?	+2	-1	0
Are there alternative causes (other than the drug) that could on their own have caused the reaction?	-1	+2	+2
Did the reaction reappear when a placebo was given?	-1	+1	0
Was the drug detected in the blood (or other fluids) in concentrations known to be toxic?	+1	0	0
Was the reaction more severe when the dose was increased, or less severe when the dose was decreased?	+1	0	0
Did the patient have a similar reaction to the same or similar drugs in any previous exposure?	+1	0	0
Was the adverse event confirmed by any objective evidence?	+1	0	0
Total score			6

## Discussion

An FDE is a type of toxicodermatitis induced by a delayed hypersensitivity reaction (type IV) that is most commonly associated with drug administration. FDE is characterized by the reappearance of erythematous-violaceous plaques at the same site upon reexposure to the triggering agent, a phenomenon referred to as "geographical memory [5]." This analysis focuses specifically on drug-induced FDE.

Drug-induced cutaneous adverse events are reported in 2-5% of hospitalized individuals. A recent review identified ibuprofen, acetaminophen, fluconazole, trimethoprim/sulfamethoxazole, and ciprofloxacin as the drugs most frequently associated with FDE [6]. Oral medications are the most common cause of FDE [2].

FDE is a delayed hypersensitivity reaction mediated by CD8+ T cells. During the first eight hours after exposure to the triggering agent, CD8+ T cells are activated and release proinflammatory cytokines, TNF-alpha, interferon-γ, perforin, and granzyme B, which damage melanocytes and basal keratinocytes. Injury to melanocytes results in the release of melanin into the dermis. This process is modulated by interleukin (IL)-10, which is produced following the activation of FoxP3+CD4+ regulatory T cells within the first 24 hours. Once the offending drug is withdrawn, the basal epidermal layer begins to regenerate, and the inflammatory cells undergo apoptosis. During this repair phase, dermal macrophages phagocytose the extravasated melanin and remain at the site, contributing to the development of residual hyperpigmentation. Additionally, basal keratinocytes secrete IL-15, promoting the development of resident memory CD8+ T cells, which are thought to play a role in the recurrence of FDE at the same anatomical location [2,7,8].

Clinical manifestations

FDE can affect any area but has a predilection for the extremities (e.g., hands and feet) and thin-skinned areas (e.g., lips, genitalia, and perianal region). Although generally asymptomatic, pruritus and pain can precede the appearance of lesions at the FDE site [2]. FDE typically begins as erythematous-violaceous oval plaques with a gray center and well-defined borders. Occasionally, a central blister is present. The most characteristic clinical finding in FDE is recurrence at the same site. During the first exposure to the drug, FDE may not appear for up to one week. After subsequent exposure to the same drug, plaques appear within 8-24 hours [9]. FDEs have multiple clinical forms. The generalized form, as seen in the present clinical case, involves more than 10% of the total body surface area or three or more anatomical sites (e.g., head, neck, anterior and posterior chest, and upper and lower extremities) [8]. FDE resolves with the discontinuation of the etiological agent, leaving residual hyperpigmentation that lasts from weeks to months [2]. With repeated episodes, the size and number of lesions may increase, resulting in severe post-inflammatory hyperpigmentation [10].

Diagnosis

A detailed inquiry into all the medications used is essential. A definitive diagnosis of FDE is obtained through an oral rechallenge or patch test. Oral rechallenge testing consists of administering a subtherapeutic dose of the suspected medication by mouth, followed by monitoring for any adverse reaction. Typically, one-tenth of the standard dose is given, and the test is performed at least two weeks after the last episode. It was previously considered the gold standard for diagnosis; however, it is now contraindicated in its generalized form [8]. A patch test is a safer and easier option. It involves applying the drug to a hyperpigmented area of skin previously affected by FDE, with normal skin used as a control. Infiltration and erythema are considered positive test results. It is very effective in identifying NSAIDs as causative agents. A biopsy can be helpful in atypical cases and those in which the diagnosis is unclear [9]. In the present case, the diagnosis was made using the Naranjo probability scale, which indicated a probable adverse drug reaction [4].

Treatment

The primary therapeutic action is discontinuation of the triggering agent, which typically leads to the resolution of the dermatosis and post-inflammatory hyperpigmentation. Currently, no effective therapeutic options can rapidly resolve post-inflammatory hyperpigmentation. As an adjunctive treatment for the inflammatory response, short cycles of topical or systemic steroids can be used, depending on the clinical extent of the lesions [8].

## Conclusions

Cutaneous adverse drug reactions constitute up to 5% of dermatological consultations. Although well-established drugs can cause FDE, this study presents a case of a generalized FDE secondary to levofloxacin, a drug that is not commonly associated with this reaction. Furthermore, the generalized variant of FDE remains underreported, highlighting the importance of documenting cases with rare clinical presentations. In this context, continuing medical education is essential, especially for the early identification by other healthcare professionals of less common variants of FDE, such as the generalized form. Given the increasing use of antibiotics in medical practice, as well as the high incidence of skin reactions associated with these medications, practitioners must be informed about potential adverse reactions. Recognizing drug reactions in a timely manner and improving their diagnosis and management contribute to patient safety and prevent potential complications.
